# A preliminary study evaluating cardiac output measurement using Pressure Recording Analytical Method (PRAM) in anaesthetized dogs

**DOI:** 10.1186/s12917-018-1392-5

**Published:** 2018-03-06

**Authors:** Angela Briganti, Flavia Evangelista, Paola Centonze, Annaliso Rizzo, Francesco Bentivegna, Antonio Crovace, Francesco Staffieri

**Affiliations:** 1 Department of Veterinary Sciences, University of Pisa, Pisa, Italy; 2 Dipartimento delle Emergenze e Trapianti di Organo, Università degli Studi di Bari “Aldo Moro,” Sezione di Cliniche Veterinarie e P.A, Bari, Italy

**Keywords:** Pressure recording analytical method, Cardiac output, Pulse contour, Dog, Anaesthesia

## Abstract

**Background:**

Haemodynamic variations normally occur in anaesthetized animals, in relation to the animal status, administered drugs, sympathetic and parasympathetic tone, fluid therapy and surgical stimulus. The possibility to measure some cardiovascular parameters, such as cardiac output (CO), during anaesthesia would be beneficial for both the anaesthesia management and its outcome. New techniques for the monitoring of CO are aimed at finding methods which are non invasive, accurate and with good trending ability, which can be used in a clinical setting. The aim of this study was to compare the Pressure Recording Analytical Method (PRAM) with the pulmonary artery thermodilution (TD) for the measurement of cardiac output in 6 anaesthetized critically ill dogs.

**Results:**

Fifty-four pairs of CO measurements were obtained with a median (range) of 3.33 L/min (0.81–7.21) for PRAM-CO and 3.48 L/min (1.41–6.56) for TD-CO. The Bland-Altman analysis showed a mean bias of 0.17 L/min with limits of agreement (LoA) of − 0.46 to 0.81 L/min. The percentage error resulted 18.2%. The 4-quadrant plot analysis showed an acceptable concordance (93%) between the 2 methods. The polar plot showed a good trending ability with the mean angular bias of 3.9° and radial LoA ± 12.1°.

**Conclusions:**

The PRAM resulted in good precision, acceptable concordance and good trending ability for the measure of CO in the anaesthetized dog, representing a promising alternative to thermodilution for the measurement of CO. Among all the pulse contour methods available on the market it is the only one that does not require any calibration or adjustment of the measurement. Further studies are required to verify the ability of this method to accurately measure cardiac output even during unstable hemodynamic conditions.

## Background

Haemodynamic monitoring represents a support tool for the assessment of the clinical status of patients, guidance of therapeutic decisions and evaluation of response to intervention, both in human and veterinary patients [1–3].

Cardiac output (CO) is a macrocirculatory measure of total body blood flow, defined as the volume of blood pumped by the heart in 1 min, and is a major determinant of DO_2_ [4]. Since most organs rely on flow rather than pressure for optimal function, measurement of CO is a pivotal component of the haemodynamic evaluation of high risk human patients, providing an indirect indication of global tissue perfusion [5]. The need to monitor this parameter in the perioperative period has led to the development of various devices and techniques. Thermodilution, through the use of pulmonary artery catheters (PAC-TD), based on the Stewart-Hamilton principle, is considered as the clinical standard method for the measurement of cardiac output in human medicine and it is often considered the reference method when other devices or measuring techniques are compared [5, 6]. However, the use of PAC-TD in clinical practice is limited, mainly because of additional costs and risks associated with pulmonary artery catheterization [7]. For this reason, in the last years less invasive procedures, such as pulse contour monitors, are under evaluation in order to find new techniques that can be used for the CO evaluation. Recent veterinary studies concluded that results obtained from the evaluated pulse contour methods [8–11] are not reliable for the CO evaluation in dogs in the clinical setting.

Pressure Recording Analytical Method (PRAM) is a pulse contour method, which estimates stroke volume and other hemodynamic parameters from the analysis of the arterial pulse waveform [12]. It is based on the principle for which the arterial blood pressure waveform results from the interaction between the systolic ejection volume and the physical characteristics of the systemic vascular system (vascular compliance, aortic impedance and peripheral arterial resistance). Stroke volume is estimated from the area under the systolic portion of an arterial pressure curve and a variable called Z. This variable indicates the dynamic impedance of the cardiovascular system, representing all the factors which oppose to the propagation of the pressure wave on the arterial tree. The variable Z is computed from the pressure curve through a proprietary algorithm on each beat [12–14]. The monitor allows a beat-by-beat assessment of CO from the arterial pressure wave, it is minimally invasive because it only needs the insertion of a regular, non-dedicated arterial catheter and doesn’t need calibration prior to clinical use. In addition to the CO, heart rate, systolic, diastolic and mean arterial pressures, pulse pressure, systolic pressure, pulse pressure variation (PPV), stroke volume (SV) and stroke volume variation (SVV) are also continuously provided by the monitor [13, 14].

PRAM has been validated for the measurement of CO in a swine model under different haemodynamic states [15, 16] and in human patients undergoing cardiac surgery [17], patients supported with a left ventricular assist device [18] and in patients supported with aortic counterpulsation after cardiac surgery [19].

The objective of this study was to evaluate the performances of the PRAM technology integrated in the MostCare® monitor compared to PAC-TD for the measurement of CO in selected cases in which dogs received a Swan-Ganz catheter for their clinical management. We hypothesized that CO measurement obtained by PRAM would be in agreement with those obtained by thermodilution.

## Methods

The study was conducted at the Section of Veterinary Clinics and Animal Production, DETO, University of Bari, Italy after the approval of the Ethical Committee (Prot. n. 48/16-DETO) and written owner consent was obtained before enrolling the dogs in the study.

### Animals

Six female dogs, affected by early stage sepsis, anesthetised for major abdominal surgical procedures, were enrolled for this study. In all dogs a Swan-Ganz catheter was placed for the monitoring of the cardiac output during anaesthesia and thereafter in the intensive care unit, based on the judgment of the clinician in charge. Patients that required administration of inotropic and/or vasoactive drugs and/or with cardiac arrhythmias were not included in the study.

Before anaesthesia all the animals were submitted to clinical and laboratory exams in order to evaluate the clinical conditions and assign the ASA Physical Status.

Breed, age, weight, ASA Physical Status and the main pathological reasons for surgery are reported in Table 1.

**Table 1:** Breed, age, weight, type of surgery and ASA status of the dogs included in the study

Case n.	Breed	Age (years)	Weight (kg)	Surgery	ASA Status
1	Golden Retriever	1	17	Pyometra	III
2	Mixed Breed	2	15	Septic peritonitis for intestinal rupture (foreign body)	IV
3	Beagle	3	17	Septic peritonitis for a rupture of an hepatic abscess	III - IV
4	German Shepherd	2	23	Pyometra	IV
5	Mixed Breed	2	14	Pyometra	III - IV
6	Mixed Breed	1	14	Septic peritonitis for anastomotic dehiscence	IV

### Anaesthetic protocol

Dogs were premedicated with methadone^1^ 0.3 mg/kg intramuscularly and were induced with propofol^2^ to effect to obtain orotracheal intubation at 30 min after the premedication. Anaesthesia was maintained with isoflurane^3^ in oxygen with an oxygen inspired fraction (FiO_2_) > 0.8. The end tidal concentration of isoflurane was maintained at 1–1.3% during all the procedure according to the patients’ requirements. During the procedure all the animals were mechanically ventilated in a volume controlled mode with a tidal volume of 10 mL/kg, a respiratory rate of 12 breaths per minute and an inspiration-expiration ratio of 1:2; the ventilator setting was modified in order to maintain the carbon dioxide end tidal (EtCO_2_) values between 35 and 45 mmHg.^4^ Surgery started at the completion of the placement of the monitoring and the surgical scrubbing. Ringer Lactate solution^5^ was administrated at a minimum of 10 mL/kg/h IV and its rate was adjusted based on the specific requirements of the patients. At the end of surgery anaesthetic drugs administration was discontinued and patients were recovered in the intensive care unit (ICU) with the appropriate support. Before surgery and in the postoperative period dogs were treated with 22 mg/kg IV of ampicillin^6^ every 8 h and enrofloxacin^7^ 10 mg/kg IV every 24 h. Moreover, methadone (0.2 mg/kg IV) was administered every 4 h. Fluid therapy, pain management, cardiovascular support, sedation and any additional treatment were based on the judgment of the clinician in charge of the case. The Swan-Ganz and arterial catheters were left in place up to a maximum of 48 h.

### Cardiac output measurements

After induction of anaesthesia patients were positioned in dorsal recumbency. A 6F 12 cm introducer was transcutaneoulsy placed in the right external jugular vein by means of the Seldinger technique, and through it a 5F Swan-Ganz pulmonary arterial catheter^8^ was advanced to the lumen of the pulmonary artery to obtain the cardiac output measurements with the thermodilution technique (TD) using a dedicated monitor.^9^ The catheter’s position was confirmed by observation of characteristic pulmonary arterial pressure waves. The computation constant for the computer was adjusted for a 5F Swan-Ganz catheter, injection volume of 5 mL, and an injectate temperature of 0–5 °C, which was measured at the injection site. Each measurement was taken with the administration of 5 mL of cold (0–3 °C) 0.9% sodium chloride^10^ over less than 3 s, the measurement was repeated for three times with 1 min intervals between each determination. Injections were done manually always by the same person, and the mean of three measurements within 10% was used. A 18 gauge × 25 mm catheter^11^ was transcutaneously positioned in the femoral artery and connected to the PRAM monitor^12^ for the evaluation of the CO.

Both the Swan-Ganz and the arterial catheters were connected to a transducer^13^ with a dedicated saline-filled line included in the kit and zeroed at the right atrial level. The accuracy of the signal was verified by a square wave test, before starting data collection.

The CO values obtained with PRAM (PRAM-CO) were recorded and stored automatically every 30 s on a personal computer. At each time of determination of CO with TD, a marker was stored on the PRAM monitor in order to detect that specific determination, a posteriori, on the PRAM data sheet. The corresponding PRAM determination was considered as the value registered at the time of the marker. The average of the values recorded for the three consecutive determinations at each time point of the study was taken as the representative result for PRAM-CO. The CO measurements were done after the placement of the catheters (T0) and thereafter every 10–15 min up to 8 measurements (T1, T2, T3, T4, T5, T6, T7, T8) for a total of 9 time points.

### Monitoring

During the procedure heart rate (HR, beats/min), systolic, diastolic, mean arterial pressures (SAP, DAP and MAP, mmHg)^12^, end-tidal carbon dioxide tension (EtCO_2_, mmHg), peripheral capillary oxygen saturation (SpO_2_, %), respiratory rate (RR, breaths/min) and oesophageal temperature (T, °C) were monitored with a multiparameter monitor^14^ and recorded every 5 min on a data sheet. Moreover, central venous pressure (CVP, mmHg), cardiac output (CO, L/min) were measured and systemic vascular resistances (SVR, dyn·s/cm^− 5^) were calculated.

### Statistics

Data were analysed for normal distribution with a Kolmogorov Smirnov test and expressed as mean and standard deviation. A one-way ANOVA for repeated measures was used to compare the clinical parameters for each recorded time; if significant, Tukey’s test was applied for post hoc comparison between the different conditions. A Spearman correlation test was used to evaluate the correlation between pairs of values (TD-CO vs PRAM-CO) and a coefficient of determination (r^2^) was calculated. *P* values < 0.05 were considered significant.

The agreement between TD-CO and PRAM-CO measurements was assessed using the Bland Altman method for multiple observations per individual [20, 21]. Mean bias (mean difference between measurements), standard deviation (SD), percentage bias (mean of 100 x [bias/(TD-CO + PRAM-CO)/2]), 95% limits of agreement (LoA; mean bias ±1.96 SD) and percentage error (100 × 1.96 SD/mean CO of both methods) were calculated. A percentage error not exceeding 30% was considered acceptable to indicate clinical reliability [22]. Precision of method (POM) of each series of triplicate CO measurements was calculated as 2 times the coefficient of error (CE) and CE was obtained dividing the coefficient of variation by the square root of number of replicates [11, 23]. The obtained POMs were used to calculate the precision of agreement (POA) [23].

Ability of the PRAM to track changes towards the TD method was evaluated by 4-quadrant plot and polar plot analysis. Sequential percentage changes in PRAM-CO were plotted on the y axis and corresponding changes in the TD-CO were visualized on x axis. The graph was then divided in 4-quadrants by the intersection of lines originated from zero in both axes. Delta CO measurements < 10% were excluded according to the bibliography [24]. The concordance was calculated as the numbers of points in the upper right and lower left quadrants, divided by the total number of points and considered as a percentage as following: > 95% good concordance, between 95% and 90% acceptable or marginal and < 90% poor concordance.

The delta CO (ΔCO) changes were then calculated as arithmetic mean of ΔCO of the tested (PRAM-CO) and reference (TD-CO) method. The polar angle was calculated as the angle of divergence of the ΔCO from the line of identity (45°) [25]. As recommended by Critchley et al. (2011) good trending ability based on polar plot analysis was defined by the mean angular bias ≤ `5° with radial LoA ≤ `30°.

For statistical test and graphic presentation different specific softwares^15^^,^^16^^,^^17^ were used.

## Results

All the enrolled animals survived to the surgical procedure and the anaesthesia. The median (range) duration of surgery and anaesthesia was 153 min (125–185) and 195 min (156–215) respectively. No complications related to the Swan-Ganz arterial catheters were reported in the records of the dogs.

No differences were detected between each recorded time for clinical parameters (Table 2).

**Table 2:** Mean values and standard deviation of measured physiological parameters of dogs for 9 time points corresponding to CO measurements

	T0	T1	T 2	T 3	T 4	T 5	T 6	T 7	T 8
HR (beats/min)	84 ± 17	78 ± 29	81 ± 24	79 ± 16	70 ± 19.	65 ± 13	72 ± 18	75 ± 21	80 ± 17
SAP (mmHg)	105 ± 16	111 ± 19	115 ± 14	118 ± 17	145 ± 31	123 ± 23	115 ± 14	114 ± 17	111 ± 18
MAP (mmHg)	74 ± 12	79 ± 16	83 ± 12	88 ± 16	114 ± 26	90 ± 25	86 ± 14	84 ± 16	81 ± 18
DAP (mmHg)	58 ± 9	64 ± 14	67 ± 11	73 ± 15	98 ± 24	79 ± 17	70 ± 13	68 ± 15	66 ± 17
CVP (mmHg)	5 ± 1	7 ± 3	6 ± 2.4	7 ± 1	8 ± 2	8 ± 2	7 ± 2	7 ± 2	6 ± 2
PRAM –CO (L/min)	2.8 ± 0.5	3.0 ± 0.5	3.4 ± 0.5	3.4 ± 0.7	3.9 ± 0.5	3.0 ± 1.2	4.1 ± 1.6	3.1 ± 0.8	3.2 ± 0.9
TD-CO (L/min)	2.8 ± 0.5	3.3 ± 0.6	3.6 ± 0.4	3.5 ± 0.9	4.1 ± 0.3	3.1 ± 0.9	4.1 ± 1.3	3.4 ± 07	3.5 ± 0.6
SV_PRAM_ (mL)	33 ± 11	46 ± 21	45 ± 16	42 ± 10	66 ± 24	42 ± 19	48 ± 16	43 ± 13	40 ± 12
SV_TD_ (mL)	33 ± 7	48 ± 22	48 ± 17	45 ± 11	63 ± 18	49 ± 17	58 ± 13	48 ± 15	45 ± 9
ETCO_2_ (mmHg)	43 ± 4	42 ± 3	43 ± 3	40 ± 6	38 ± 6	44 ± 6	45 ± 6	43 ± 5	44 ± 5
SPO_2_ (%)	95 ± 1	95 ± 2	96 ± 1	95 ± 1	97 ± 1	97 ± 1	96 ± 1	96 ± 1	97 ± 1
T (°C)	36.9 ± 0.6	36.6 ± 0.7	36.4 ± 0.9	36.4 ± 0.9	35.9 ± 0.6	35.7 ± 0.4	35.4 ± 0.5	35.6 ± 0.4	35.5 ± 0.5
RR (breaths/min)	*14* ± 3	*14* ± 3	*14* ± 3	*14.* ± 2	*15* ± 3	*16* ± 2	*16* ± 2	*16* ± 2	*16* ± 2

*HR* heart rate, *SAP* systolic arterial pressure, *MAP* mean arterial pressure, *DAP* diastolic arterial pressure, *CVP* central venous pressure, *PRAM-CO* cardiac output measurements obtained by PRAM, *TD-CO* cardiac output measurements obtained by pulmonary artery thermodilution, *SV*_*PRAM*_ stroke volume measurements obtained by PRAM, *SV*_*TD*_ stroke volume measurements obtained by pulmonary artery thermodilution, *EtCO*_*2*_ end-tidal carbon dioxide tension, *SpO*_*2*_ peripheral capillary oxygen saturation, *T* temperature, *RR* respiratory rate

**Fig. 1:**
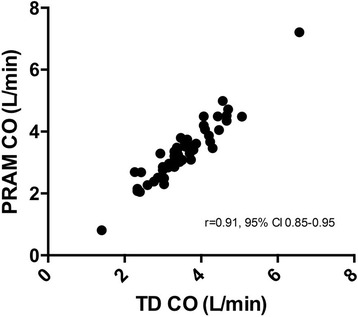
Plot of PRAM-CO values (y axis) and TD-CO values (x axis) with Spearman correlation test results

Fifty-four pairs of CO measurements were obtained (Fig. 1).

**Fig. 2:**
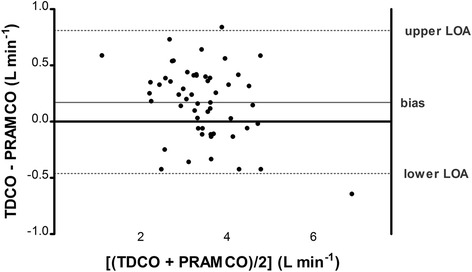
Bland Altman plot for 54 pairs of CO measurements. Light grey line: mean bias; dashed lines: upper and lower limits of agreement

The median and range of the CO values were 3.33 L/min (0.81–7.21) for the PRAM and 3.48 L/min (1.42–6.56) for the TD. The Bland-Altman analysis showed a mean bias of 0.17 L/min with LoA of − 0.46 to 0.81 L/min. The percentage error resulted 18.2% (Table 3). A Bland-Altman plot is shown in Fig. 2. The POM of the TD-CO was 9.2% and POM of PRAM-CO was 8.8%. POA expected if the reference technique was compared to itself (POA _REFxREF_) resulted 13%; POA of test versus reference technique (POA _TESTxREF_) resulted 12.7%.

**Table 3:** Results of Bland Altman Analysis for 54 pairs of CO measurements

	TD-CO vs PRAM-CO
mean bias (L min^−1^)	0.17
SD of bias (L min^−1^)	0.32
mean percentage bias (%)	6.38
upper LOA (L min^− 1^)	0.81
lower LOA (L min^− 1^)	−0.46
percentage error (%)	18.2

**Fig. 3:**
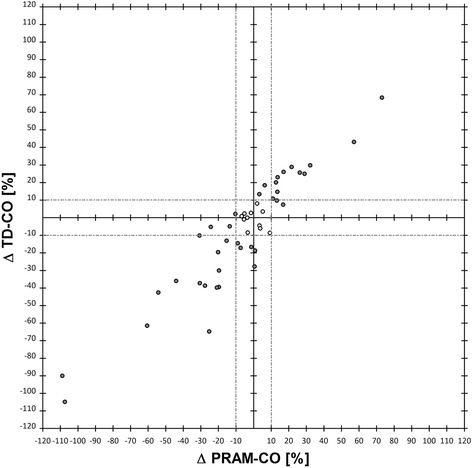
Four-quadrant plot. Sequential percentage changes (∆CO) of PRAM-CO (Y axis) and of TD-CO (x axis). The central grey zone represents the exclusion area that contains ∆CO < 10%. Upper right and lower left quadrant contain concordant ∆CO, upper left and lower right quadrant contain discordant ∆CO

**Fig. 4:**
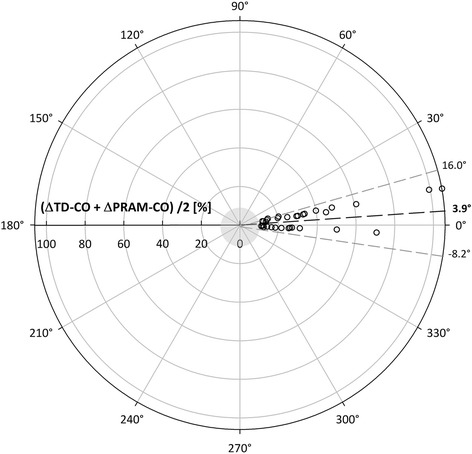
Polar plot of ∆CO expressed as polar coordinates (radius and polar angle). The central grey circle represents the exclusion zone (mean ∆CO < 10%). The black dashed line represents the mean polar angle of 3.9° (mean angular bias), light gray dashed lines represent the radial LoA (± 12.1°). Sixteen data points were excluded from analysis because they fell in the exclusion zone

The 4-quadrant plot analysis (Fig. 3) showed an acceptable concordance (92%) between the 2 methods (Table 4). The polar plot showed a good trending ability with the mean angular bias of 3.9° with radial LoA ± 12.1° (Table 3 and Fig. 4).

**Table 4:** Results of 4-quadrant plot and polar plot

	TDCO vs PRAMCO
Measurements < 10% (n)	11
Measurements > 10% in the right quadrants (n)	34
Measurements > 10% in the wrong quadrants (n)	3
Total measurements > 10% (n)	37
Concordance rate (%)	92
Mean angular bias (°)	3.9
Radial limits of agreement (°)	± 12.1

Concordance rate was calculated as: 100 × (data points in correct quadrant and > 10% CO)/data points > 10% CO. Concordance rates are considered to be good when above 95%, acceptable when between 90 and 95% and poor when below 90%. In the polar plot a mean angle with the horizontal axis < 5° and radial limits of agreement RLOA (= 95% confidence interval) < 30° are considered to indicate good trending ability

## Discussion

This is the first study in which the PRAM technology is compared to the thermodilution method in the canine species. These preliminary findings showed a close agreement between thermodilution and PRAM. The PRAM method tends to underestimate CO compared to thermodilution measurements with a mean bias of 0.17 L/min (and a mean percentage bias of 6.38%). The percentage error is a statistical measure of agreement between the two methods [24]. A limit of 30% of the percentage error value has been recommended as an objective criterion to define clinical acceptability of a new method compared to the reference method [22]. The percentage error in this study, calculated from the Bland-Altman plot, resulted 18%, largely acceptable for the aforementioned limit of percentage error. This value is consistent to percentage errors found in other validation studies of CO measuring methods, and even better performing than other studies involving the validation of PRAM in man [12, 18, 19, 26]. The limitation to the commonly utilization of the 30% cut-off value for the percentage error is due to the fact that precision of thermodilution technique can vary and, in case of rigorously applied techniques, or when CO is derived from more than 1 measurement, a low PE of the reference method can compensate for higher PE of the tested technique. For this reason the most recent studies, evaluating new techniques for the cardiac output comparison, put in evidence the necessity to calculate the specific precision of each used method [27]: both the one considered the reference method (POM_REF_) and the new one studied (POM_TEST_). With this calculation it was possible to evaluate the precision of each method (POM) employed in the present study, both for the reference (TD-CO: 9,2%) and the tested method (PRAM-CO: 8,8%). POM itself can not be used to compare the two techniques because it describes the similarity of a method’s repeated measurements for one and the same true value [27]. The main problem in using a POM in CO studies is that the CO itself is not a constant true value, but it can rapidly change over time depending on several factors such as heart rate, stroke volume and mean arterial pressure. For simplicity, in this kind of studies, it is assumed that for each time point cardiac output is fairly constant during the multiple measurements obtained by both techniques [11]. POM_REF_ and POM_TEST_ can be used to calculate the POA, which describes the variability in the agreement or disagreement between methods [27]. Usually POA between the studied technique and the reference technique (POA_TESTxREF_) is compared to the POA that would be expected if the reference technique was compared to itself. POA_REFxREF_, defined as estimation of the POA when the reference technique is compared to itself, is an artificial POA, but can be used to define the acceptable percentage error of a specific study. In the present study the POA_REFxREF_ resulted 13% and POA_TESTxREF_ resulted 12.7%. In case of the POM of the studied technique and the reference technique were the same also the POA_REFxREF_ and POA_TESTxREF_ would be the same [27] and the tested technique would be accepted as in our case. It is fundamental to keep in mind that POM only describes deviations among repeated measurements of a true value, while the trueness describes the overall deviation of these measurements from that value, that in case of cardiac output measurement it is impossible to acquire [27].

In the validation process of a new technique, Bland-Altman analysis is the main instrument to assess if the new method agrees with the reference method and to which extent [28]. However it is important to point out that this statistical tool only addresses agreement and doesn’t provide information about how reliably the new method detects changes in the measured values in time (trending ability); thus Bland-Altman analysis alone is not sufficient to determine if a new technique is clinically acceptable in comparison to a reference technique [28, 29]. To overcome this issue, other statistical tools are available. Trending ability analysis with concordance rate calculation and four quadrant plots [25, 28] and polar plots, with the calculation of the polar angle and radial limits of agreement [25, 29], can be used to assess the trending ability of the technique to be validated. In this study both the 4-quadrant analysis and the polar plot have been done in order to assess the concordance and the trending ability of the PRAM technique. The concordance resulted acceptable (92%) and the trending ability good (mean radial angle of 3.9° and radial LoA ± 12.1°).

Both for the 4 quadrant plot and for the polar plot, a central exclusion zone with data points ≤ 10% was identified and data inside it were excluded. Those data were considered to increase the signal-to-noise ratio, [24, 25] adding no information to the assessment of trending ability of the technique and representing very little clinical impact.

A recent evaluation of pulse contour methods in dogs has been published [8]. The authors evaluated PiCCO and PulseCO (both externally calibrated pulse contour methods, respectively with transpulmonary thermodilution and with pulmonary artery thermodilution), as well as transpulmonary thermodilution, all compared to pulmonary artery thermodilution. Since the assessment of agreement and of trending ability with the same statistical tools allows the comparison of different studies [28] we can compare our results to those obtained by Kutter and colleagues. They show a poor trending ability of PiCCO and PulseCO compared to pulmonary artery thermodilution with low concordance rates (respectively 77% and 74%), and an unacceptable percentage error (respectively 47% and 42%), far above the 30% criterion defined by Critchley (2010) for acceptability. In our study the concordance and the trending ability of PRAM resulted superior to all the techniques evaluated by Kutter and colleagues with a mean radial angle lower than 5° and all the measurements under ±30°.

Another recent study [28] evaluated the use a calibrated pulse contour analysis, PiCCO (calibrated with transpulmonary thermodilution) during pharmacological interventions on vascular tone (intravenous phenylephrine and nitroprusside) in dogs. The authors concluded that the use of PiCCO is limited in dogs when abrupt changes in peripheral vascular resistances occur, reporting low concordance (63%) and poor trending ability (mean radial angle 38°) compared to pulmonary artery thermodilution.

The Flotrac/Vigileo system, a pulse contour method, which needs an input of demographic data for calibration, has been evaluated in dogs in comparison to pulmonary artery thermodilution [9]. In light of a very high percentage bias between the two techniques (110%) and a high percentage error, far beyond the limits of acceptance (162%), the authors conclude that the use of this monitor is not recommended for measuring CO in dogs [9]. Moreover authors did a post-hoc trending ability analysis and poor trending ability with a concordance rate of 86% was found together with a mean polar angle of 9° as well as inadequately wide radial limits of agreement of ±48° (Kutter and Bektas, personal communication). The algorithm which underlies the functioning of FloTrac/Vigileo takes into account human demographic data which might not apply correctly to the cardiovascular system of dogs [9].

Pulmonary artery thermodilution is a technique which requires some time to obtain one single measurement. In this time frame the true value of CO can experience variations and therefore for each single measurement there is an intrinsic error, which can be reduced by averaging a certain number of single measurements. For TD-CO determination it has been recommended to obtain the mean of three consecutive measurements of CO, which results in a precision of 6% within the true value with a 95% confidence interval, and allows a reliable detection in CO changes [30, 31].

For this reason a monitor which can rapidly detect changes in CO might be more suitable for use in critical and surgical patients, or in goal directed therapy, contexts in which it is crucial to detect fast hemodynamic changes. Pulse contour methods estimate SV from the arterial wave line and can perform a beat-to-beat estimation of CO.

If our results will be confirmed by more standardized and controlled studies, the PRAM technology could represent a promising alternative to thermodilution for the measurement of CO in dogs, both for clinical and research purposes. The PRAM method only needs a pressure waveform signal detected by an arterial catheter in a peripheral artery connected to a dedicated monitor, and among all the pulse contour methods available on the market it is the only one that does not require any calibration or adjustment of the measurement according to pre-obtained data [14]. The issue of calibration is overcome by PRAM, which obtains information about the arterial system elastic and mechanical properties in vivo, directly from the arterial waveform, for every beat [13]. Indeed pulse contour methods calculate SV from the ratio of the area under the arterial pressure curve and a variable called Z, which denotes the relationship between pressure and flow. Z takes into account physical properties of the cardiovascular system, which are closely interrelated, such as arterial impedance, arterial compliance and the peripheral arterial tree resistance. All these physical properties need to be evaluated simultaneously for a correct estimation of SV from the pressure wave [13]. In calibrated pulse contour methods, Z is computed starting from already existing data (Vigileo) or it is obtained from an external calibration method (PiCCO, PulseCO) and its value is applied over several SV estimations, even if the mechanical and elastic properties of the arterial system may change with time. PRAM, instead, directly estimates Z from the curve at every pulsation, considering both the systolic and diastolic contributions of the curve to the estimation of SV [13, 32]. Therefore the way that the variable Z is obtained may be the key difference between pulse contour methods that explains why PRAM has a superior performance in dogs, showing a good agreement and trending ability when compared to pulmonary artery thermodilution.

On the other side, PRAM has some limitations related to the need to detect dicrotic notch at every beat. If the monitor fails to correctly detect the dicrotic notch at each beat, the calculations are incorrect and CO measures might be artifactual. In our study the femoral artery has been catheterised, whereas other locations most commonly used in clinical patients, as dorsal pedal or dorsal metatarsal arteries, could be more influenced for the vascular elasticity and the dicrotic notch could not be as clear as in our results. The occurrence of arrhythmia or bradycardia is a typical scenario in which the monitor can mis-detect the notch and therefore return erroneous readings. However, the position of the dicrotic notch is highlighted on the screen at every beat, and the clinician can modify the settings on the PRAM monitor to adjust the dicrotic notch position if the quality of the detection is evaluated to be poor. Another issue is the necessity to have a correct damping of the arterial line: assessment of the damping is important since an over- or under-damped signal can generate an artefact which modifies the area under the curve and therefore affects the estimation of SV [32].

There is no information about the use of PRAM during acute changes in vascular resistance in dogs. Garofalo and colleagues (2016) report that when nitroprusside was infused intravenously in dogs, the dicrotic notch of the arterial waveform was blunted and difficult to detect by PiCCO; while the dicrotic notch was always detectable during a phenylephrine intravenous infusion. This is probably due to the dispersion of energy in the vascular tree during a vasodilated state, which decreases the magnitude of reflected backward waves from the peripheral arterial system to the heart, a phenomenon that could lead to failure of the device to detect the end of the systolic portion of the wave and to overestimate SV, artifactually increasing the area under the curve of the systolic portion [11].

The number of the cases is an important limitation of this study. Moreover, animals that required administration of vasoactive and/or inotropic drugs were excluded in order to avoid additional biases that could not have been controlled in a clinical setting. Accordingly, our observations were limited to more stable cardiovascular conditions. In the light of these encouraging results, further experimental and clinical studies are needed to determine whether PRAM has a good agreement and trending ability during cardiovascular instability and under the influence of drugs that alter the peripheral vessel tone, thus confirming the clinical reliability of this technique.

## Conclusion

PRAM resulted in good precision, acceptable concordance and good trending ability for the measure of CO in the anaesthetized dog with a clinically stable hemodynamic status. As a minimally-invasive pulse contour method it seems to be very promising. Further studies are needed to assess the capacity of this method to be accurate even during instable hemodynamic conditions.

## Abbreviations

PAC: With pulmonary artery catheter
TD: Thermodilution
CE: Coefficient of error
CO: Cardiac output
CVP: Central venous pressure
DO_2_: Systemic oxygen delivery
EtCO_2_: Carbon dioxide end tidal
FiO_2_: Oxygen inspired fraction
HR: Heart rate
LoA: Limit of agreement
PE: Percentage error
POA: Precision of agreement
POM: Precision of method
PRAM: Pressure Recording Analytical Method
RR: Respiratory rate
SAP, DAP, MAP: Systolic, diastolic, mean arterial pressure
SD: Standard deviation
SpO_2_: Peripheral capillary oxygen saturation
SV: Stroke volume
SVR: Systemic vascular resistances
TD: Thermodilution
VO_2_: Oxygen consumption
ΔCO: Delta cardiac output

## Footnotes

1 Semfortan 10 mg/mL, Dechra S.r.l

2 Propovet 10 mg/mL^,^ Zooetis

3 Vetflurane, 1000 mg/g, Virbac Animal Health

4 Servo 900D, Siemens Elma, Sweden

5 B.Broun Vet Care 500 ml

6 Vetamplius 10 g 50 mL, Fatro Spa

7 Baytril 10%, Bayer Spa

8 Swan-Ganz, Edwards Lifesciences, Irvine, Calif

9 Vigilance®, Edward Lifescience Corporation

10 B.Broun Vet Care 500 ml

11 Neo Delta Ven®, Delta Med, Medical Services

12 Most Care®, Vytech, Padova, Italy

13 Transpac®, IV Monitoring Kit, Icu Medical

14 Siemens, SC 6002XL Patient Monitor

15 GraphPad Prism 6.1, GraphPad Software, Inc.

16 Microsoft ® Office Excel 2003, Microsoft Corporation

17 SigmaPlot for Windows, Version 14.0, Systat Software, Inc.
